# The interaction of love in clinical care and its connection with burnout of health professionals

**DOI:** 10.1192/j.eurpsy.2022.770

**Published:** 2022-09-01

**Authors:** A.–.L. Batiridou, E. Dragioti, S. Mantzoukas, E. Kotrotsiou, M. Gouva

**Affiliations:** 1 University of Ioannina, Laboratory Of Psychology Of Patients, Families And Health Professionals, Ioannina, Greece; 2 University of Ioannina, Laboratory Of Psychology Of Patients, Families And Health Professionals)., Ioannina, Greece; 3 University of Ioannina, Nursing, Ioannina, Greece

**Keywords:** health professionals, burnout, Love, Care

## Abstract

**Introduction:**

The phenomenon of love in the clinical field, in other words, the practical love through specific features, is the “ultimate investment” of the well-being, both to the patient and to the health professional.

**Objectives:**

The aim of this research study is to investigate the role of love and its connection with burnout in the context of clinical professional care.

**Methods:**

The study was cross-sectional and was conducted from September 2020 to February 2021 at the Nursing Department of University of Ioannina, Greece. The sample of the present study was determined to be health professionals, both sex from all over Greece. The research tools which were used in the quantitative study were: 1) Socio-demographic questionnaire, 2) Measurement of social representations of love and 3) Maslach Burnout Inventory - MBI.

**Results:**

The results of the present quantitative research showed that gender, religion, family environment, place of residence, years of work and job position of health professionals affect the love and compassion they can show and offer to their patients, and the love is related to the level of burnout they experience (p <0.05), in the context of clinical occupational care.

**Conclusions:**

In conclusion, love, its traits and expression of the feelings of health professionals, determine the level of clinical care and the burnout of health professionals.

**Disclosure:**

No significant relationships.

